# Inhibitory effect of microRNA-608 on lung cancer cell proliferation, migration, and invasion by targeting *BRD4* through the JAK2/STAT3 pathway

**DOI:** 10.17305/bjbms.2019.4216

**Published:** 2020-08

**Authors:** Weigang Xu, Dapeng Sun, Yanqin Wang, Xinlin Zheng, Yan Li, Yu Xia, Ya’nan Teng

**Affiliations:** 1 Department of Respiratory Medicine, The Affiliated Weihai Second Municipal Hospital of Qingdao University, Shandong, China; 2 Department of Health Examination, The Affiliated Weihai Second Municipal Hospital of Qingdao University, Shandong, China

**Keywords:** microRNA-608, *BRD4*, lung cancer, migration, invasion

## Abstract

Lung cancer is the leading cause of cancer-related mortality around the world. This malignancy has a 5-year survival rate of 21%, because most of the patients are diagnosed in the middle or late stage of the disease when local metastasis and tumor invasion have already progressed. Therefore, the investigation of the pathogenesis of lung cancer is an issue of crucial importance. MicroRNAs (miRNAs) seem to be involved in the evolution and development of lung cancer. MicroRNA-608 is likely to be downregulated in lung cancer tissues. Regarding this, the current study involved the determination of the fundamental mechanism of microRNA-608 in the development of lung cancer. Based on the results of quantitative reverse transcription polymerase chain reaction (RT-qPCR), the expression level of microRNA-608 was downregulated in 40 lung cancer tissues, compared to that in the adjacent normal tissues. The results of dual-luciferase reporter assay revealed that bromodomain-containing protein 4 (*BRD4*) was the direct target of microRNA-608. Accordingly, the lung cancer tissues had an elevated expression level of *BRD4*, in contrast to the adjacent normal tissues. The results of Cell Counting Kit 8 assay demonstrated that the high expression of microRNA-608 notably restrained lung cancer cell proliferation. The scratch wound and transwell assays showed that the upregulation of microRNA-608 suppressed the migration and invasion of lung cancer cells. Finally, the western blot assay showed that in the microRNA-608 mimics group, the expression levels of *BRD4*, p-JAK2, p-STATA3, CD44, and MMP9 were significantly decreased, compared with those in the negative control miRNA mimics group. Our results indicate that high expression of microRNA-608 inhibits the proliferation, migration, and invasion of lung cancer cells by targeting *BRD4* via the JAK2/STAT3 pathway.

## INTRODUCTION

Lung cancer is a malignant pulmonary tumor, accounting for a high rate of cancer-related mortality across the world [1]. This malignancy is characterized by uncontrolled cell growth and metastasis that may spread from the lung into the nearby tissues or other parts of the body [2]. According to the statistics, lung cancer caused at least 1.6 million deaths worldwide in 2012 [3]. Overall, less than 18% of American people diagnosed with lung cancer have a five-year survival [4]. However, the outcomes of this disease are even worse for the people living in developing countries. Therefore, the identification of the mechanisms of lung cancer development is a matter of urgency.

MicroRNAs (miRNAs) are small non-coding RNA molecules, containing about 22 nucleotides [5]. These RNAs function in RNA silencing and post-transcriptional regulation of gene expression. Based on the evidence, microRNAs are involved in many cellular processes, such as cell proliferation [6], apoptosis [7], migration [8], invasion [9], epithelial-mesenchymal transition [10], and differentiation [11]. Moreover, the dysregulation of microRNAs also contributes to the development and metastasis of many cancers, such as lung [12], breast [13], gastric [14], ovarian [15], and liver cancers [16].

Recent data are indicative of the significantly lower expression of microRNA-608 in lung cancer tissues than in the adjacent normal tissues [17]. Furthermore, another report also revealed that the upregulation of microRNA-608 could increase cell death in A549 and SK-LU1 cells [18]. The results of the aforementioned studies are indicative of the involvement of microRNA-608 in the progression of lung cancer. However, the relationship between microRNA-608 and lung cancer and the molecular mechanism remain to be revealed.

With this background in mind, the current study was performed to verify the expression of microRNA-608 and its potential target gene, namely bromodomain-containing protein 4 (*BRD4*), in lung cancer tissues and adjacent normal tissues. After confirming that *BRD4* was the direct target of microRNA-608, the effect of microRNA-608 on lung cancer cell proliferation was investigated using a Cell Counting Kit 8 (CCK-8) assay. Scratch wound and transwell assays were performed so as to unveil the influences of microRNA-608 on the capabilities of lung cancer cell migration and invasion. Finally, the effect of microRNA-608 on *BRD4* and related proteins was verified by western blot assay. Our study illustrated that microRNA-608 may restrain the abilities of proliferation, migration, and invasion of lung cancer cells by targeting *BRD4* through the JAK2/STAT3 pathway.

## MATERIALS AND METHODS

### Specimens

This study was conducted on 40 pairs of human lung cancer tissues and adjacent normal tissues, supplied from the Affiliated Weihai Second Municipal Hospital of Qingdao University, Weihai, China, between May 2014 and February 2016. Based on pathological examinations, all patients were diagnosed with lung cancer, without receiving any kinds of treatment prior to the research (Table 1). After excision, the collected specimens were immediately preserved in liquid nitrogen at - 80°C. Prior to the onset of the experiment, written informed consent was obtained from all patients or their relatives. The research project was approved by the Ethics Committee of the Affiliated Weihai Second Municipal Hospital of Qingdao University.

**TABLE 1:**
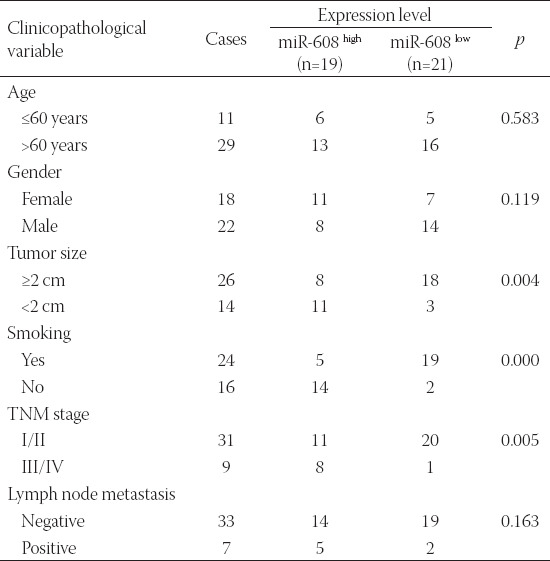
Cliniopathological characteristics of 40 patients with lung cancer

### Dual-luciferase reporter assay

A549 human lung cancer cells (Gefen Biotechnology. Co., LTD, Shanghai, China) were transfected with the wild type *BRD4* 3’untranslated region (UTR) and mutant *BRD4* 3’UTR, which were cloned into p-miR-GLO plasmid (Fenghuishengwu, Changsha, China), together with microRNA-608 mimics or negative control (NC) mimics using the Lipofectamine™ 2000 (Thermo Fisher Scientific, Inc., Waltham, MA, USA). After incubation for 4 h at 37°C, the luciferase activities were measured by means of a dual-luciferase reporter gene assay kit (Yeasen Biotechnology Co., Ltd, Shanghai, China).

### Cell culture and transfection

The A549 cell line was prepared and cultivated in RPMI-1640 medium (Solarbio Technology Co., Ltd, Beijing, China) supplemented with 10% fetal bovine serum (FBS; Thermo Fisher Scientific, Inc., Waltham, MA, USA) and 1% antibiotics (100 U/ml penicillin and 100 µg/ml streptomycin). The cells were incubated at 37°C in a humid atmosphere with 5% CO_2_.

In order to determine the transfection efficiency of microRNA-608, the cells were first divided into three different groups: i) control group; ii) negative control miRNA (miR-NC) mimics group (i.e., cells transfected with miRNA-NC mimics); and iii) miR-608 mimics group (i.e., cells transfected with microRNA-608 mimics). The cells were seeded on six-well plates, with the density of 5×10^5^ cells/well. Subsequently, they were transfected with microRNA-NC mimics or microRNA-608 mimics using the Lipofectamine™ 2000 (Thermo Fisher Scientific, Inc., Waltham, MA, USA) following the manufacturer’s instructions.

The microRNA-608 mimics and microRNA-NC mimics were purchased from the GenePharma Co., Ltd (Shanghai, China). In order to further determine the transfection efficiency of *BRD4*, the cells were divided into another three different groups: i) control group (i.e., untreated cells); ii) pcDNA3.1 group (i.e., blank plasmid); and iii) pcDNA3.1-*BRD4* (i.e., cells transfected with pcDNA3.1-*BRD4*). The cells were seeded on six-well plates at a density of 5×10^5^ cells/well. The pcDNA3.1 was supplied from the Thermo Fisher Scientific, Inc. (Waltham, MA, USA).

### RNA extraction and quantitative reverse transcription polymerase chain reaction (RT-qPCR) analysis

Total RNA was extracted by TRIzol (Invitrogen; Thermo Fisher Scientific, Inc., Waltham, MA, USA) from the lung cancer tissues and A549 cells, according to the manufacturer’s protocol. Subsequently, 2 µl RNA was reverse transcribed into complementary DNA (cDNA) using the All-in-One First-Strand cDNA Synthesis Kit (GeneCopoeia Inc., USA) following the manufacturer’s protocol. The isolation of microRNAs from the lung cancer tissues and A549 cells was accomplished via the EasyPure^â^ miRNA Kit (TransGen Biotech Co., Ltd, Beijing, China) based on the instructions. In the next stage, miRNAs were reverse transcribed to cDNAs using the miRNA First-Strand cDNA Synthesis (Sangon Biotech Co., Ltd, Shanghai, China). Afterward, RT-qPCR analysis was performed by means of the TaqMan MicroRNA RT kit (Applied Biosystems; Thermo Fisher Scientific, Inc., Waltham, MA, USA) following the instructions. Briefly, 2 µl cDNA, 10 µl SYBR GREEN I reagent (Thermo Fisher Scientific, Inc., Waltham, MA, USA), 1 µl primers, and dd-H_2_O were mixed together for PCR reaction. The reaction procedure included denaturation at 95°C for 3 min and then at 94°C for 30 sec, annealing at 52°C for 30 sec, extension at 72°C for 30 sec (each procedure was performed for 30 cycles), and then final extension at 72°C for 10 min. During the whole process, U6 small nuclear RNA and glyceraldehyde-3-phosphate dehydrogenase (GAPDH) were used as endogenous controls for miRNA and mRNA, respectively. All procedures were conducted in triplicate, and relative expression was calculated using the 2-^ΔΔCt^ method.

### Measurement of cell proliferation rate by Cell Counting Kit 8 (CCK-8) assay

The transfected cells were reseeded onto a 96-well plate at the density of 2×10^5^ cells/well in a novel RPMI-1640 medium (Solarbio Technology Co., Ltd, Beijing, China) 24 h post-transfection. The medium was incubated for 48 h at 37°C and in 5% CO_2_. In the next stage, 10 µl CCK-8 solution (Solarbio^â^ Life Sciences, Beijing, China) was added to the medium 12 h, 24 h, and 48 h later. After incubation for another 10 min, the absorbance at 490 nm was detected with a spectrophotometer (Thermo Fisher Scientific, Inc., Waltham, MA, USA).

All experiments were performed in four groups: i) control group (i.e., untreated cells); ii) miR-NC mimics group (i.e., cells transfected with microRNA-NC mimics); iii) miR-608 mimics group (i.e., cells transfected with microRNA-608 mimics); and iv) miR-608 mimics+*BRD4* group (i.e., cells transfected with microRNA-608 mimics, along with pcDNA3.1-*BRD4*).

### Measurement of cell migratory ability by wound scratch assay

In order to determine the migratory capability of lung cancer cells in different groups, a scratch wound assay was performed. The A549 cells were seeded on a six-well plate at a density of 3×10^5^ cells/well. Then, a straight line was drawn with a sterilized 10-µl pipette tip on the surface of the well. Afterward, serum-free medium was added and cultivated for 24 h at 37°C and 5% CO_2_. The distances between the wound sides were captured in the same fields at the baseline and 24 h later using an inverted microscope (×200). All the procedures were operated in triplicate, and the percent of the wound was analyzed.

### Measurement of cell invasive ability by transwell assay

A 10-µm pore Tranwell (Xinshengyuan Biomart Ltd, Beijing, China), along with Matrigel (Qcbio Science & Technologies Co., Ltd, Shanghai, China), was applied to verify the lung cancer cell invasive ability. The A549 cells were put in the top chamber with 200 µl serum-free medium. Furthermore, 500 µl complete culture medium and 10% FBS (Thermo Fisher Scientific, Inc., Waltham, MA, USA) were added to the bottom chamber.

The Transwell was washed with PBS twice 24 h after transfection, and then stained with 0.1% crystal violet (Baomanbio Biomart Co, Shanghai, China) for 15 min at 37°C. The cells that failed to invade to the bottom chamber were carefully removed with a sterilized cotton swab. The cells that invaded the bottom chamber were imaged and counted under an inverted microscope (×200). All the procedures were performed in triplicate.

### Investigation of related protein expression by western blot assay

The extraction of total protein from the lung cancer tissues and A549 cells was accomplished by means of the Minute™ Protein Extraction Kits (Invent Biotechnologies, Inc., Plymouth, Minnesota, USA) supplemented with protease inhibitor cocktail (×100; Thermo Fisher Scientific, Inc., Waltham, MA, USA). The Bradford method was used to verify the concentration of proteins in the supernatants. GAPDH acted as the internal control. Afterward, 10 µl protein was separated with 10% sodium dodecyl sulfate–polyacrylamide gel electrophoresis (SDS-PAGE, Thermo Fisher Scientific, Inc., Waltham, MA, USA) and transferred to a polyvinylidene fluoride membrane (PVDF; Renoldbio Biomart Ltd, Suzhou, China).

The PVDF membrane was then blocked with 50 ml 5% BSA solution and incubated at 4°C with rabbit anti-*BRD4* (1:1000; ab128874; Abcam, Cambridge, MA, USA), rabbit anti-janus kinase 2 [JAK2] (1:1000; ab68269; Cobioer Biocart Ltd, Nanjing, China), rabbit anti-phospho (p)-JAK2 (1:1000; ab32101; Abcam, Cambridge, MA, USA), rabbit anti-signal transducer and activator of transcription 3 [STAT3] (1:1000; ab68153; Abcam, Cambridge, MA, USA), rabbit anti-p-STAT3 (1:2000; ab76315; Abcam, Cambridge, MA, USA), rabbit anti-CD44 (1:2000; AB157107; Abcam, Cambridge, MA, USA), rabbit anti-matrix metallopeptidase 9 [MMP9] (1:1000; ab38898; Abcam, Cambridge, MA, USA), and rabbit anti-GAPDH (1:2500; AB9485; Abcam, Cambridge, MA, USA) overnight. Subsequently, IgG H&L (HRP; ab7090; Abcam, Cambridge, MA, USA) secondary antibody was added and incubated at 37°C for another 2 h. After the membranes were washed with Tris-buffered saline/Tween 20 three times, the chemiluminescence reagents (Abcam, Cambridge, MA, USA) were added. The relative protein expression levels of *BRD4*, CD44, and MMP9 were normalized to the internal control GAPDH, while p-JAK2 and p-STAT3 were normalized to the internal JAK2 and STAT3. The autoradiography was quantified using the ImageJ Software (version 1.49; National Institutes of Health, Bethesda, MD, USA).

### Statistical analysis

In order to analyze the data, IBM SPSS Statistics for Windows, Version 19.0. (IBM Corp., Armonk, NY, USA) was used. The data were presented as mean ± standard deviation. In addition, Student’s t-test was applied to calculate the differences between two groups. One-way analysis of variance (ANOVA), followed by NewmanKeuls posthoc analysis, was also employed to distinguish the differences among three or four groups. A p-value less than 0.05 was considered statistically significant.

## RESULTS

### Expression level of microRNA-608 in lung cancer tissues and adjacent normal tissues

**FIGURE 1:**
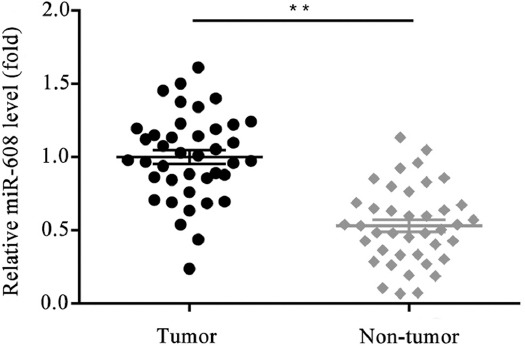
Comparison of the relative expression level of microRNA-608 in lung cancer tissues and adjacent normal tissues (**p < 0.01 vs. control).

In order to determine the biological role of miR-608 in lung cancer tissues, RT-qPCR analysis was applied to detect the expression levels of miR-608 in lung cancer and adjacent normal tissues, obtained from 40 patients with lung cancer. As shown in Figure 1, the expression level of microRNA-608 was markedly reduced in the lung cancer vs. control group (*p* < 0.01).

### *BRD4* as a direct target of microRNA-608 in A549 lung cancer cell line

**FIGURE 2:**
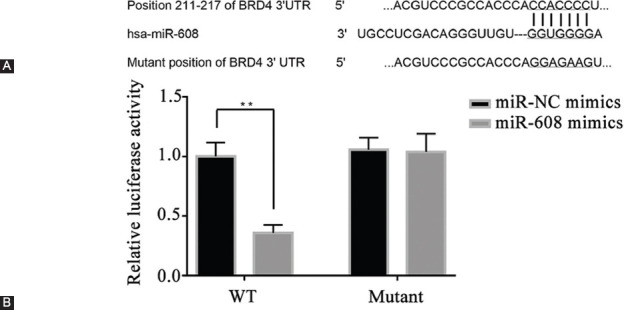
MicroRNA-608 directly targets *BRD4*. (A) The sequence alignment of the paired site of 3’-UTR of microRNA-608 and *BRD4*; (B) the activity of luciferases in different groups (**p < 0.0 vs. NC mimics group). WT: Wild-type; miR-608: MicroRNA-608; UTR: Untranslated region; *BRD4*: Bromodomain-containing protein 4; NC: Negative control.

Based on the online bioinformatic tool TargetScan, *BRD4* was predicted to be the potential target of microRNA-608. Therefore, a dual-luciferase reporter assay was implemented to verify the association between microRNA-608 and *BRD4*. As shown in Figure 2A, the 3’-UTR of the *BRD4* gene was found to contain the binding sequences of microRNA-608, suggesting *BRD4* as a probable downstream target of microRNA-608. A549 cells transfected with microRNA-608 mimics and wild-type *BRD4* 3’UTR plasmid had a significant decrease in luciferase activity; no variation in luciferase activity was observed in the mutant-type *BRD4* 3’UTR plasmid-transfected cells (*p* < 0.01; Figure 2B).

### Expression level of *BRD4* in lung cancer tissues and adjacent normal tissues

**FIGURE 3:**
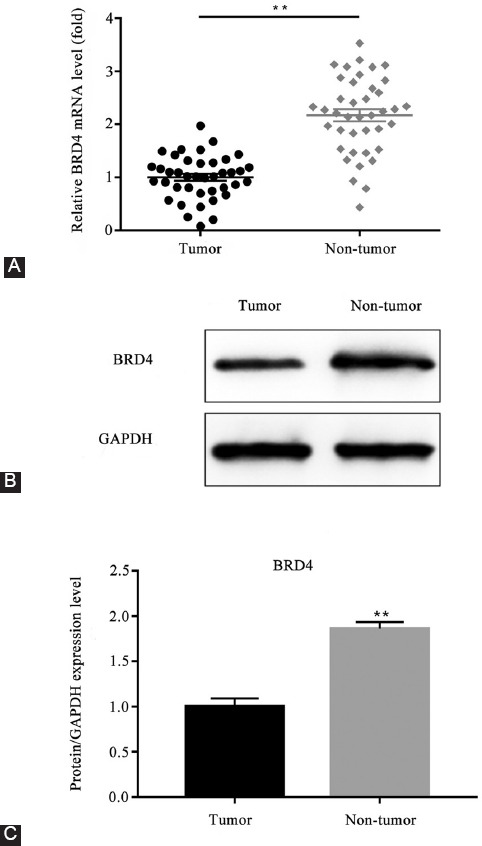
Comparison of the relative expression level of *BRD4* in lung cancer tissues and adjacent normal tissues. (A) The relative expression level of *BRD4* mRNA in lung cancer tissues and adjacent normal tissues; (B-C) the protein expression level of *BRD4* in lung cancer tissues and adjacent normal tissues (**p < 0.01 vs. control group). *BRD4*: Bromodomain-containing protein 4; GAPDH: Glyceraldehyde 3-phosphate dehydrogenase.

As *BRD4* was revealed to be the direct target of microRNA-608, the expression level of *BRD4* in lung cancer tissues and adjacent normal tissues was measured. As the results exhibited, the expression level of *BRD4* mRNA was highly elevated in the control group, compared to the lung cancer group (*p <* 0.01; Figure 3A). The protein expression by western blot assay showed the same trend of change (*p <* 0.01; Figure 3B and C).

### Transfection efficiency of microRNA-608 and *BRD4*

**FIGURE 4:**
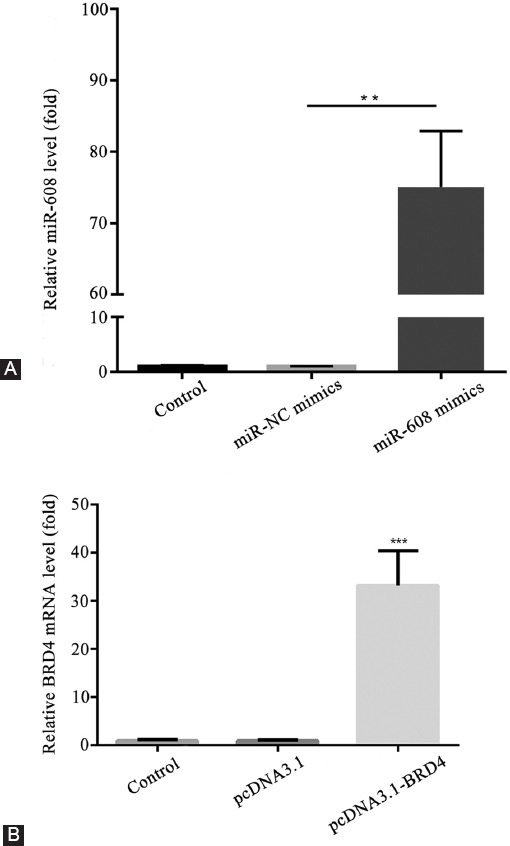
Transfection efficiency of microRNA-608 and *BRD4*. (A) The relative expression level of microRNA-608 in different groups (**p < 0.01 vs. miR-NC mimics group); (B) the relative expression level of *BRD4* mRNA in different groups (***p < 0.001 vs. pcDNA3.1 group). MiR-608: MicroRNA-608; *BRD4*: Bromodomain-containing protein 4; NC: Negative control.

The transfection efficiency was confirmed by RT-qPCR assay. As shown in Figure 4A, the expression level of microRNA-608 was prominently elevated in the microRNA-608 mimics group, compared with that in the microRNA-NC mimics group (*p <* 0.01). The results also revealed that the expression level of *BRD4* mRNA was markedly increased in the pcDNA3.1-*BRD4* group, compared to that in the pcDNA3.1 group (*p* < 0.001; Figure 4B).

### Upregulated microRNA-608 significantly decreased lung cancer cell proliferation rate

**FIGURE 5:**
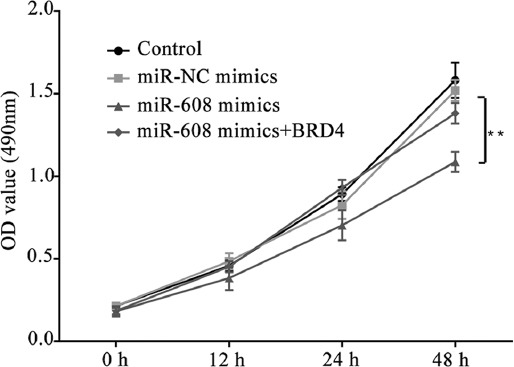
Results of Cell Counting Kit 8 assay showing the reduction of lung cancer cell proliferation rate by upregulated microRNA-608 (optical density value in different groups; **p < 0.01 vs. miR-NC mimics group). MiR-608: MicroRNA-608; BRD4: Bromodomain-containing protein 4; NC: Negative control.

To investigate the effect of microRNA-608 on lung cancer cell proliferation the CCK-8 assay was performed. As shown in Figure 5, compared to the microRNA-NC mimics group, the proliferation rate was decreased in the microRNA-608 mimics group, which was rescued by the transfection of pcDNA3.1-*BRD4* (*p* < 0.05).

### Upregulated microRNA-608 remarkably reduced lung cancer cell migration and invasion

**FIGURE 6:**
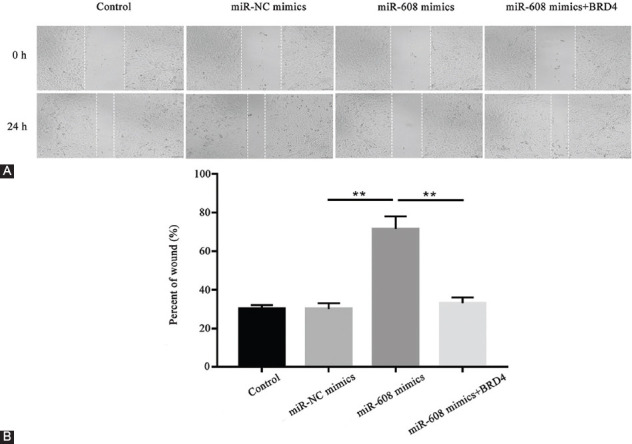
Results of scratch wound assay showing the restraint of the migratory capability of lung cancer cells by upregulated microRNA-608. (A) The implementation of wound scratch to determine the capability of cell migration in different groups; (B) the corresponding data of A are presented (the cell migration rate was calculated by migration distance/original width; **p < 0.01, miR-608 mimics vs. miR-NC mimics group; miR-608 mimics + *BRD4* vs. miR-608 mimics group). MiR-608: MicroRNA-608; *BRD4*: Bromodomain-containing protein 4; NC: Negative control.

In order to validate the impact of microRNA-608 on the lung cancer cells, scratch wound and transwell assays were conducted in triplicate. With regard to the scratch wound assay (Figure 6A and B), in contrast to microRNA-NC mimics, the percent of the wound was significantly elevated in the microRNA-608 mimics group, and this was significantly decreased by the overexpression of *BRD4* in the microRNA-608 mimics+*BRD4* group (*p* < 0.01).

**FIGURE 7:**
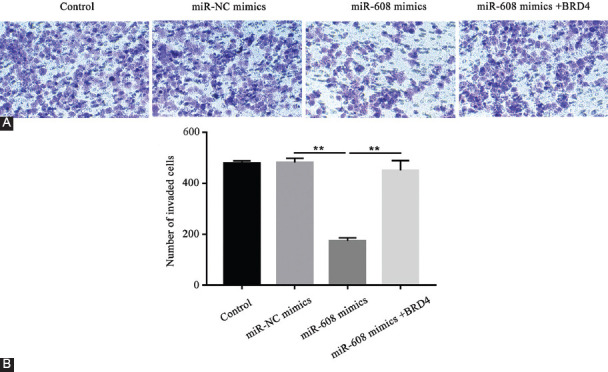
Results of transwell assay showing the restraint of the invasive capability of lung tumor cells by upregulated microRNA-608. (A) The implementation of transwell assay to determine the capability of lung cancer cell invasion in different groups; (B) the number of invaded cells in different groups (**p < 0.01, miR-608 mimics vs. miR-NC mimics group; miR-608 mimics+*BRD4* vs. miR-608 mimics group). MiR-608: MicroRNA-608; *BRD4*: Bromodomain-containing protein 4; NC: Negative control.

The transwell assay demonstrated that, compared with microRNA-NC mimics, the number of invaded cells notably decreased in the microRNA-608 mimics group, which was reversed by the transfection of pcDNA3.1-*BRD4* in the microRNA-608 mimics+*BRD4* group (*p* < 0.01; Figure 7A and B). In other words, the migration and invasion abilities of lung cancer cells were dramatically restrained in the microRNA-608 mimics group, and this was partially abolished by the *BRD4* overexpression.

### Effect of microRNA-608 on the expression levels of *BRD4*, JAK2, p-JAK2, STAT3, p-STAT3, CD44, and MMP9

The expression levels of *BRD4*, JAK2, p-JAK2, STAT3, p-STAT3, CD44, and MMP9 were measured so as to reveal the underlying mechanism of microRNA-608 and *BRD4*.

**FIGURE 8:**
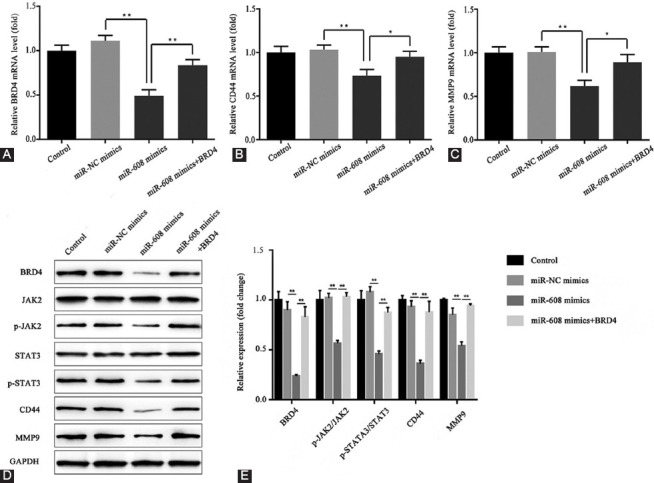
Effect of microRNA-608 on the expression levels of *BRD4*, JAK2, p-JAK2, STAT3, p-STAT3, CD44, and MMP9. (A) The relative expression level of *BRD4* mRNA in different groups; (B) the relative expression level of CD44 mRNA in different groups; (C) the relative expression level of MMP9 mRNA in different groups; (D) the protein expression levels of *BRD4*, JAK2, p-JAK2, STAT3, p-STAT3, CD44, and MMP9 in different groups; (E) the corresponding data of D are presented (**p < 0.01; miR-608 mimics vs. miR-NC mimics group; miR-608 mimics+*BRD4* vs. miR-608 mimics group). MiR-608: MicroRNA-608; BRD4: Bromodomain-containing protein 4; NC: Negative control; GAPDH: Glyceraldehyde-3-phosphate dehydrogenase; JAK2: Janus kinase 2; p: Phosphorylated; STAT3: Signal transducer and activator of transcription 3; MMP9: Matrix metallopeptidase 9.

The results of the RT-qPCR assay demonstrated that, compared with the microRNA-NC mimics, the mRNA levels of *BRD4*, CD44, and MMP9 significantly decreased in the microRNA-608 mimics group, which was reversed significantly by the overexpression of *BRD4* in the microRNA-608 mimics+*BRD4* group (*p* < 0.01; Figure 8A-C).

The western blot assay showed that, compared with the microRNA-NC mimics, the expression levels of *BRD4*, p-JAK2, p-STAT3, CD44, and MMP9 declined in the microRNA-608 mimics group, which was reversed by *BRD4* overexpression in the microRNA-608 mimics+*BRD4* group (*p* < 0.01; Figure 8D-E).

## DISCUSSION

MicroRNA-608 is reported to inhibit cell proliferation in several types of cancers, such as human hepatocellular carcinoma [19], bladder cancer [20], colon cancer [21], and chordoma [22]. Furthermore, it was also demonstrated that microRNA-608 could inhibit the migration and invasion of glioma stem cells [23] and even act as a prognostic marker in hepatocellular carcinoma [24]. With regard to lung cancer, microRNA-608 was found to exert tumor-suppressive effect in lung adenocarcinoma by directly targeting the macrophage migration inhibitory factor [25].

Another report pointed out that microRNA-608 could inhibit human lung adenocarcinoma via the regulation of AKT2 [26]. In other words, microRNA-608 was considered to be the tumor suppressor, which could restrain cell proliferation and metastasis in many cancers. In line with previous reports, our results revealed that microRNA-608 might restrain cell proliferation, migration, and invasion in lung cancer cells. Our study further discovered the relationship and mechanism between microRNA-608 and its putative target *BRD4*.

Using the dual-luciferase reporter assay, we confirmed that *BRD4* is the direct target of microRNA-608. *BRD4* was upregulated in the lung cancer tissues, while the inhibition of *BRD4* by microRNA-608 mimics suppressed lung cancer cell proliferation, migration, and invasion. On the other hand, the overexpression of *BRD4* could restore the above effects. According to the literature, *BRD4* might promote cell proliferation in pancreatic ductal adenocarcinoma [27], hepatocellular carcinoma [28], and colorectal cancer [29].

*BRD4* was also proved to be involved in the progression of metastasis. For instance, *BRD4* could induce cell migration and invasion in hepatocellular carcinoma [30] and breast cancer [31]. In the analyzed cases of lung cancer, a high level of *BRD4* was considered to be correlated with a poor prognosis of non-small cell lung cancer (NSCLC) and promotion of NSCLC proliferation, migration, and invasion [32]. Taken together, *BRD4* appears to be an oncogene in various cancers and is associated with the progression of cancer metastasis. However, the effects of microRNA-608 mimics on related signaling pathways and molecules involved in metastasis are yet to be investigated.

In the literature, the JAK2/STAT3 pathway was frequently associated with cancer metastasis [33-35]. With regard to lung cancer, the JAK2/STAT3 pathway was also found to mediate the process of cancer metastasis [36]. Therefore, we put forward the speculation that the knockdown of metastasis-related protein *BRD4* could mediate the JAK2/STAT3 pathway and suppress the lung cancer cell migration and invasion. However, the underlying mechanisms between *BRD4* and the JAK2/STAT3 pathway remained to be clarified.

The CD44 is a multifunctional cell surface molecule involved in cancer cell proliferation [37] and metastasis [38]. Hence, based on the western blot results, we predicted that the knockdown of *BRD4* could decrease the expression level of CD44, jointly inhibiting the lung cancer cell migration and invasion. MMPs play an important role in tissue remodeling associated with various pathological processes, such as morphogenesis [39], angiogenesis [40], tissue repair [41], and metastasis [42]. MMP-9 is also assumed to be important in cancer cell metastasis [43].

Furthermore, previous studies demonstrated that the knockdown of *BRD4* could decrease the expression level of MMP9 protein, thereby restraining the migratory and invasive abilities of squamous cell carcinoma [44] and hepatocellular carcinoma [30]. Consistent with the results presented in the literature, our findings revealed that the upregulation of microRNA-608 could reduce the expression levels of *BRD4*, p-JAK2, p-STAT3, CD44, and MMP9.

## CONCLUSION

Based on the findings of the present study, it can be concluded that microRNA-608 may inhibit cell proliferation, migration, and invasion of lung cancer by targeting *BRD4* through the JAK2/STAT3 pathway.
